# Supplementary material for: Topological data analysis of human brain networks through order statistics
Source: PLoS One. 2023 Mar 13;18(3):e0276419. doi: 10.1371/journal.pone.0276419 (PMC10010566; doi:10.1371/journal.pone.0276419)
Supplement: Attachment — Submitted filename: ReviewTDABrainStats.pdf [file pone.0276419.s001.pdf]

Review: Topological Data Analysis of Brain Networks Through Order Statistics

The authors develop order statistics to compute expected Betti curves for the graph filtration of weighted networks. Specifically, they propose the expected topological loss (ETL) as a test statistic. They showcase the ETL on random graphs with 10 nodes and compare it to the maximum gap statistics, the area under the Betti-0 curve, the bottleneck distance, the Gromov-Hausdorff distance, and the Kolmogorov-Smirnov distance. They then apply the ETL to weighted networks with 116 nodes created from resting state fMRI data. Using the approach on the data, they find a statistically significant topological difference between male and female brain networks.

The manuscript is well written and addresses an interesting question. We recommend that the manuscript be accepted with minor revisions (see comments below):

- p4: It would be helpful to give the reader an intuition about what you mean by independent cycles. Similarly, a brief explanation how to compute the total number of cycles would be helpful.
- p4: Since you mention that you consider the edge weights as the filtration values rather than, for example, relative filtration steps or a normalised filtration - how would such a change in filtration affect your developed statistics?
- p4: You only consider edges and nodes in your filtration giving only barcodes in H0 and H1. Could your approach be generalised to a clique filtration, which has in the past also been applied in neuroscience contexts?
- p8: Since slight changes in weight distribution significantly change the topology of the network, is the distribution of edge weights in the data comparable enough between different subjects to motivate group level summaries? Does this aspect change when moving from resting state to task-based fMRI data?
- p7: Figure 2 is referred to very late in the text. An earlier reference would be helpful for the reader.
- p14: What rationale is behind choosing networks that are very small compared to the data? I.e. 10 nodes versus 116?
- p17: Has this difference in male and female brain networks been observed in this type of data before?
