# Supplementary material for: Topological data analysis of human brain networks through order statistics
Source: PLoS One. 2023 Mar 13;18(3):e0276419. doi: 10.1371/journal.pone.0276419 (PMC10010566; doi:10.1371/journal.pone.0276419)
Supplement: Attachment — Submitted filename: Response.2022.08.06.pdf [file pone.0276419.s002.pdf]

Response Letter

Soumya Das, D. Vijay Anand, and Moo K. Chung

We thank the associate editor and the reviewer for constructive comments that improved the revision substantially. We addressed every comment and question very carefully in the revision. The edited parts as well as the new materials are colored in blue in the main manuscript.

# Reviewer 1-1 I cannot recommend this article for publication based on the technical methods used. The analysis is described as an application of topological data analysis (TDA), but the actual tools used are more correctly described in terms of classical hierarchical clustering. Specifically, the authors develop their methods only in the context of the 1D graph, not any enrichment into a simplicial complex. Since they can study only 0D and 1D homology in this setting, and they discard the barcode in favor of lists of birth/death times, it turns out that they are using an impoverished version of hierarchical single linkage clustering, as I will make clear below. Explicitly, they rely on the classical graph theory fact that, if one removes the (n choose 2) edges of a complete graph in some order, at exactly (n-1) steps, the number of components will increase. Thus, the rest will decrease the number of circuits.

Response: *We have added additional explanations as follows.*

The brain networks are traditionally represented and analyzed as a graph, a 1-skeleton consisting of only nodes and edges [Tzourio-Mazoyer et al., 2002, Desikan et al., 2006, Hagmann et al., 2007, Fornito et al., 2016, Arslan et al., 2018]. The main focus of functional brain network analysis is quantifying and modeling the pairwise interaction between brain regions, which is usually called the *effective connectivity* [Park et al., 2018, Horwitz et al., 2005, Schlösser et al., 2003, Dirkx et al., 2016]. Thus, we limited our algebraic representation of brain networks to graphs. Compared to the vast body of studies analyzing brain networks as graphs, modeling them as higher order simplicial complexes are only few [Giusti et al., 2016, Reimann et al., 2017, Petri et al., 2014, Tadić et al., 2019]. We used the graph filtration, which iteratively builds nested subgraphs of the original graph in a hierarchical manner [Lee et al., 2011]. Currently, this is the most often used filtration in analyzing brain networks due to its simplicity.

Elaborating the comment given by the reviewer, we have added the following paragraph.

During the graph filtration, edges are deleted one at a time from the lowest edge weight to the highest. The deletion of an edge disconnect the graph into at most two. Thus, the number of connected components (β_0) stays the same or increases at most by one. Euler characteristic χ of the graph is given by [Adler et al., 2010]

$$\chi = \beta_0 - \beta_1 = \# \text{ of nodes} - \# \text{ of edges}. \quad (1)$$

Thus the change of Euler characteristic $\Delta\chi$ over the filtration is given by

$$\Delta\chi = \Delta\beta_0 - \Delta\beta_1 = 1, \quad (2)$$

where the change of β_0 is $\Delta\beta_0 = 0$ or 1. Subsequently the change of β_0 is $\Delta\beta_1 = -1$ or 0. The number of cycles decrease at most by 1 [Chung et al., 2019a].

# Reviewer 1-2 As the authors track only the locations of these changes – the "birth" and "death" times of bars in the 0D and 1D barcode in this very simple case – as they note, the steps which increase the number of components are the complement of those that decrease the number of circuits. However, this tells us that the data contained in the list of 1D death times is entirely determined by the list of 0D birth times; we simply take the complement in the ordered list (1..(n choose 2)). Thus, the 1D Betti curve they describe contains no information not available using the 0D Betti curve and the size N of the network. The information they retain is the data of the heights of splitting of components in the merge tree, but not the data of which branches are attached to which. As such, the statistic being used in the paper is effectively the expected heights of branching in a random merge tree. I am not sure if this is well-studied in the literature, but it seems like a careful review of the relevant work – some of it stretching back decades – should be performed before the authors can claim novelty, and in any case that prior work by the community that studies such objects should be cited. Further, the authors should be careful to cite primary sources for TDA methods; many of the ideas they discuss here first appear in early work of e.g. Kahle on homology of random graphs, and various groups' early work on statistical TDA for data analysis.

Response: *Following reviewers suggestions, we have added additional explanations. Although there are few papers recently coming out connecting barcodes to merge trees, it is still not well studied and without many applications beyond 0D. Accommodating the reviewer's comments, we added the following paragraphs.*

Unlike the Rips and Morse filtrations, the birth and death values are actually the 0D and 1D persistences in graph filtrations [Songdechakraiwut and Chung, 2020b]. During the filtration, once a connected component is born at b_i, it never dies implying an infinite death value. On the other hand, all the cycles are considered to be born at $-\infty$ and dies at death value d_i. Ignoring $-\infty$ and ∞, 0D persistence is completely characterized by birth values b_i while 1D persistence is completely characterized by death values d_i.

β_0 and β_1 curves are related through equation (2). Once we know the shape of β_0 curve, we exactly know the shape of β_1 curve. However, there is no known theory that can determine if $\Delta\beta_0$ is 0 or 1 at this moment. Thus, one may treat $\Delta\beta_0$ and $\Delta\beta_1$ as Bernoulli random processes and model them statistically. This is the main motivation of the paper. Alternatively one may approach the problem through the heights of branching points in random merge trees for 0D persistence [O'Neil et al., 1996, Sears and Ramakrishnan, 2012, Morozov and Weber, 2013, Liu et al., 2016, Nigmetov and Morozov, 2019, Samardzic et al., 2020]. However, merge trees have not been well investigated for 1D persistence except few studies [Obayashi, 2018].

As suggested, we also performed additional literature review on random graphs.

The random graphs have been investigated by many authors [Gilbert, 1959, Erdös and Rényi, 1961, Newman et al., 2001, Janson et al., 2011, Bobrowski and Kahle, 2018]. A graph whose features related to nodes and edges are determined in a random fashion is called a random graph. The theory of random graphs lies at the intersection between graph theory and probability theory. They are usually described using a probability distribution or a stochastic process that generates them [Bollobás and Béla, 2001, Frieze and Karoński, 2016]. The homology of random graphs have been studied studied by Kahle in particular. [Kahle, 2007] investigated the connectivity of neighborhood complex of a random graph. [Kahle, 2011] studied the expected topological properties of Rips complexes built on randomly distributed points in $\mathbb{R}^d$. [Kahle and Meckes, 2013] worked on the central limit theorem for Betti numbers of random simplical complexes. Random graphs are often encountered in graphical models, which build probabilistic models on the conditional dependency structures between nodes [Jordan, 1999, Bishop, 2006]. However, topology is rarely investigated in graphical models.

As suggested, we also performed additional literature review on statistical TDA.

This requires the statistical version of TDA [Fasy et al., 2014, Bubenik, 2015]. [Fasy et al., 2014] worked on computing confidence bands through bootstraps. [Bubenik, 2015] introduced persistent landscapes which lies in a vector space, where the sample mean and variance can be computed and thus enable a proper statistical inference. [Kwitt et al., 2015] worked on the hypothesis testing on the Gaussian kernel smoothing on persistent diagrams Analogous to the persistent barcodes, we also have their stochastic versions referred as the expected persistent barcodes. However, computing them requires complex theoretical constructs and they are generally approximated [Gayet and Welschinger, 2014, Salepci and Welschinger, 2018, Wigman, 2021].

# Reviewer 1-3 In addition, the authors claim that their statistic is useful for reducing the computational burden of topological data analysis. However, on networks of the size the authors are studying, modern software will easily compute persistence diagrams up through degrees three or four on a standard laptop, and much larger networks can be studied on even moderately powerful servers. Thus, the examples in the paper do not serve to demonstrate utility of the method.

Response: *We have added additional explanations in Discussion section. The main computational bottleneck is actually caused by resampling that requires the computation of the test statistic over half million permutations.*

The proposed graph filtration computes the barcodes in $\mathcal{O}(p \log p)$, which is significantly faster than Rips filtrations. In traditional Rips filtrations, the computational complexity grows rapidly with the number of simplices [Topaz et al., 2015]. With p nodes, the size of the k-skeleton grows as p^{k+1} and the computational run time is $\mathcal{O}(p^{3k+3})$ [Solo et al., 2018, Chung et al., 2020]. Compared to the graph filtration, the Rips filtration constructed using Ripser package [Bauer, 2021] is about 8 times slower in a computer. On top of that, we also need to compute the Wasserstein distance between persistent diagrams [Sharathkumar and Agarwal, 2012]. The Wasserstein distance computation requires expensive optimization process involving $\mathcal{O}(p^6)$ run-time [Edmonds and Karp, 1972, Kerber

et al., 2017].

Our algorithm exploits the geometric structure of the graph filtration, resulting in the persistence diagram representation in the form of 1-dimensional sorted scalar values. Thus, the proposed method computes he Wasserstein distance in $\mathcal{O}(p \log p)$ bypassing multitude of computational bottlenecks. For resampling based statistical inference such as the permutation test, the computational bottleneck is caused by repreatedlby computing the test statistic over the random permutations of group labels at least half million times [Chung et al., 2019b]. This is impractical if the Wasserstein distance for the Rips filtrations has to be used for 400 networks and then the whole computation has to be done repeatedly half million times. The development of scalable computation will have a significant impact in resampling based statistical inference.

# Reviewer 1-4 Finally, the authors repeatedly claim that this information completely characterizes the "topology" of a graph. If by this they mean that the branching heights of a merge tree are a complete invariant of weighted complete graphs, we can see that this is untrue simply by noting that there are multiple non-isomorphic merge trees with the same list of branch heights: consider a tree with two branches where both attach to the trunk versus one where the shorter branch attaches to the longer.

Response: *Following the suggestion, we have added additional explanations and toned down our statements. We meant that the* $0D$ *and* $1D$ *barcodes "together" characterize the topology of a graph; not just the "branching heights of a merge tree," which is only the* $0D$ *barcode [Morozov and Weber, 2013]. We have clarified this in the revision.*

When we increase the filtration value ϵ, either one new connected component appears or one cycle disappears. Once a connected component is born, it never dies implying an infinite death value. On the other hand, all the cycles are considered to be born at $-\infty$. Therefore, we simply ignore the infinite death values of the connected components and the negative infinite birth values of the cycles and build the computation framework based on only the birth (death) values of the connected components (cycles) [Songdechakraiwut and Chung, 2020b]. Also, the number of connected components (or cycles) is non-decreasing (or non-increasing) as ϵ increases. Subsequently, the 0D barcode is given by a set of increasing birth values:

$$B(G) : \epsilon_{b_1} < \cdots < \epsilon_{b_{m_0}},$$

and the 1D barcode is given by a set of increasing death values:

$$D(G) : \epsilon_{d_1} < \cdots < \epsilon_{d_{m_1}}.$$

By tracing the birth values of connected components and the death values of cycles *together*, we can characterize the topology of the graph.

# Reviewer 1-5 There are several places in the manuscript where careful editing would improve clarity. In particular, the subscripted indices i_a and j_a used in the definition of the order statistic are used for pages without addressing how one would go about finding them, or even acknowledging that the authors have a method for doing so. In addition, the above-

Figure 1: The independent cycles are explained through this figure in the mainbody.

mentioned claim about characterizing topology is unclear without further explanation, as are a range of claims made about TDA.

Response: *Sorry for confusion. We added the explanation on the subscripts in the order statistic as follows.*

Let $\mathcal{G}(p, \boldsymbol{W})$ be a random graph and its sorted *random* edge weights be

$$W_{(1)} < W_{(2)} < \cdots < W_{(q)},$$

where the subscript (i) indicates the ith smallest edge weight. For instance, $W_{(1)} = \min_{1 \leq i \leq q} W_i$ is the smallest edge weight while $W_{(q)} = \max_{1 \leq i \leq q} W_i$. is the largest edge weight. Order statistics can be formulated by modeling indices $_{(i)}$ using random permutations while the actual edge weights are fixed nonrandom quantity. However, traditionally in order statistics, the indices $_{(i)}$ themselves are not considered as random but fixed [Conover, 1980, Gibbons and Chakraborti, 2011]. They simply indicates the order the random variables are indexed. Only what is in the ith variable is considered as random. In this study, we will follow the traditional convention in order statistics.

# Reviewer 2-1 p4: It would be helpful to give the reader an intuition about what you mean by independent cycles. Similarly, a brief explanation how to compute the total number of cycles would be helpful.

Response: *We have added a clarification on independent cycles and how to compute the total number of cycles.*

A *cycle* or loop is a path that starts and ends at the same node but no other nodes in the path are overlapping. The cycles we identify using birth-death decomposition are essentially algebraically independent of each other and hence form a basis for cycles [Songdechakraiwut and Chung, 2022]. In binary graph G_{W_3} in Figure 1, there is one cycle consisting of edges $W_{(4)}$, $W_{(5)}$ and $W_{(6)}$. The cycle can be algebraically represented as $[W_{(4)}] + [W_{(5)}] + [W_{(6)}]$ with the convention of putting clockwise orientation along the edges. In the complete graph G_0, there are three cycles consisting of $[W_{(4)}] + [W_{(5)}] + [W_{(6)}]$, $-[W_{(5)}] + [W_{(3)}] + [W_{(2)}]$ and $[W_{(4)}] + [W_{(3)}] + [W_{(2)}] + [W_{(6)}]$. They are linearly dependent in a sense that the cycle consisting of four nodes can be written as the sum of the two other smaller cycles:

$$[W_{(4)}]+[W_{(3)}]+[W_{(2)}]+[W_{(6)}] = ([W_{(4)}]+[W_{(5)}]+[W_{(6)}])+(-[W_{(5)}]+[W_{(3)}]+[W_{(2)}]).$$

Thus, there are only two algebraically independent cycles in $G_{-\infty}$. We can represent any cycle in a graph as a linear combination of independent cycles.

The total number of algebraically independent cycles is the 1st Betti number β_1, which is equivalent to the number of death values of cycles. For a complete graph with p nodes, the total number of edges is $p(p-1)/2$. In a graph filtration, the total number of birth values of connected components equals to the number of edges $p - 1$ in the maximum spanning tree.

From the birth-death decomposition, the remaining edges contribute to the death values of cycles. The remaining number of edges are [Songdechakraiwut and Chung, 2020b]

$$m_1 = \frac{p(p-1)}{2} - (p-1) = \frac{(p-1)(p-2)}{2}.$$

# Reviewer 2-2 p4: Since you mention that you consider the edge weights as the filtration values rather than, for example, relative filtration steps or a normalized filtration - how would such a change in filtration affect your developed statistics?

Response: *The following additional explanations are added in Discussion section.*

We can also use different filtration schemes such as relative filtration [Murai et al., 2001] or normalized filtration that scales filtration values between 0 and 1 [Kannan et al., 2019]. As long as the order of sorted edge weights are not changed, they will not affect the statistical results. The Wasserstein distance we used is defined on the sorted edge weights. As long as we do not change the value of edge weights, the statistical results will not change.

# Reviewer 2-3 p4: You only consider edges and nodes in your filtration giving only barcodes in H0 and H1. Could your approach be generalized to a clique filtration, which has in the past also been applied in neuroscience contexts?

Response: *The following additional explanations are added in Discussion.*

Our methodology is based on the graph filtration, which gives both 0D and 1D persistence as monotonic 1D functions of birth and death values only. On the other hand, the clique filtration [Petri et al., 2013], does not produce monotone persistence or barcodes and the proposed method is not directly applicable [Stolz et al., 2016, Stolz et al., 2017, Ignacio and Darcy, 2019, Nguyen et al., 2020, Piangerelli et al., 2020, Giunti et al., 2021]. Our method is applicable to any filtration that provides monotone persistence or Betti curves.

# Reviewer 2-4 p8: Since slight changes in weight distribution significantly change the topology of the network, is the distribution of edge weights in the data comparable enough between different subjects to motivate group level summaries? Does this aspect change when moving from resting state to task-based fMRI data?

Response: *We have addressed the issue in the Discussion as follows.*

Graph filtrations produce the monotone birth and death values over filtrations. Since our birth and death values are exactly edge weights, the slight changes in edge weight distribution will correspondingly change the birth and death values slightly. Since the expected topological loss (ETL) is sum of differences of birth and death values, it will also change correspondingly. This is the very reason which assures that our method can successfully discriminate topological group differences whenever there is a difference in edge distributions between two groups.

The monotone Betti curves usually follow S-shaped curves similar to the pattern of cumulative distribution functions. The pattern of S-shaped Betti curves do not change drastically even if different dataset is used as long as they are weighted graphs. To demonstrate

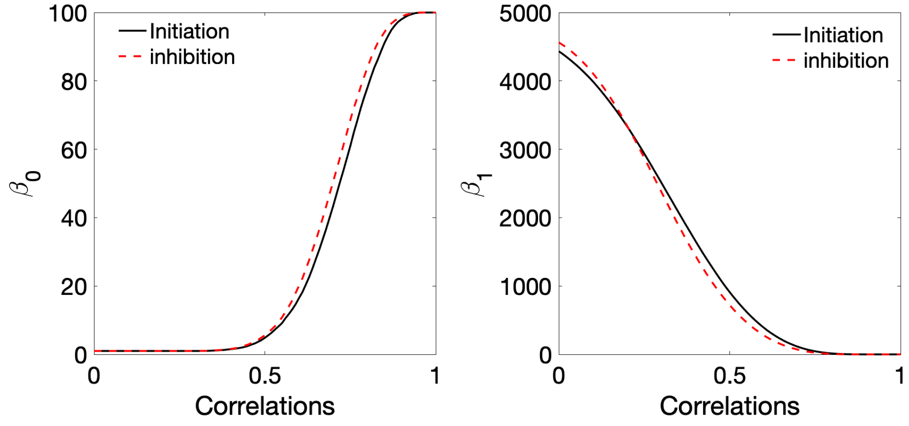

Figure 2: The average Betti curves of obtained from the graph filtration on correlation matrices computed separately for the inhibition (go) and initiation (no-go) blocks of fMRI time series in cognitive aging study [Rieck et al., 2021a, Rieck et al., 2021b].

this, we plotted Betti curves for task-fMRI. Figure 2 shows the Betti curves for task-fMRI, where cognitive inhibition was measured using go/no-go paradigm in 144 subjects on 100 brain regions [Rieck et al., 2021a, Rieck et al., 2021b]. The correlation matrices were computed separately for the inhibition (go) and initiation (no-go) blocks of fMRI time series. The monotonic Betti curves show almost identical pattern of Betti curves in rs-fMRI networks in our study.

# Reviewer 2-5 p7: Figure 2 is referred to very late in the text. An earlier reference would be helpful for the reader.

Response: Figure 2 is now referenced early and it is now labeled as Figure 1.

# Reviewer 2-6 p14: What rationale is behind choosing networks that are very small compared to the data? I.e. 10 nodes versus 116?

Response: *Following the suggestion, we also added simulation setting for large network with* $p = 100$ *networks and added 3 new Tables 1, 2 and 3.*

We investigated the performance of both small ($p = 10$) and large ($p = 100$) network settings. Small networks may not yield complex cyclic structures often present in large networks. However, the overall conclusions are the same regardless of the size of networks.

# Reviewer 2-7 p17: Has this difference in male and female brain networks been observed in this type of data before?

Response: *We did literature and responded as follows. They are included at the end of Result section.*

The sex differences of resting state functional networks were previously investigated. There is known sex difference in the parietal region involved in spatial ability [Koscik et al., 2009]. [Xu and Lindquist, 2015] reported sex differences in the left parietal, precentral and postcentral regions. The sex difference is also reported in the left rolandic operculum [Rubin et al., 2017]. The previous rs-fMRI studies mainly focused on brain region specific

Table 1: The average p-values obtained using the ETL statistic for various pairs of distributions considered for drawing edge weights of networks. Here, the columns 6 networks, 8 networks, 10 networks, and 12 networks indicate the number of networks that we considered for both the groups. The p-values smaller than 0.01 indicate that our method can identify network differences at a 99% confidence level.

Distribution	6 networks	8 networks	10 networks	12 networks
$\text{Beta}(1,1)$ vs. $\text{Beta}(5,2)$	0.0021	9.0000×10^{-5}	2.0000×10^{-5}	0.0000
$\text{Beta}(1,1)$ vs. $\text{Beta}(1,5)$	0.0018	8.0000×10^{-5}	2.0000×10^{-5}	1.0000×10^{-5}
$\text{Beta}(5,2)$ vs. $\text{Beta}(1,5)$	0.0022	1.2000×10^{-4}	0.0000	0.0000
$\text{Beta}(1,1)$ vs. $\text{Beta}(1,1)$	0.6430	0.2844	0.3308	0.2665
$\text{Beta}(5,2)$ vs. $\text{Beta}(5,2)$	0.7882	0.8828	0.5559	0.9319
$\text{Beta}(1,5)$ vs. $\text{Beta}(1,5)$	0.1526	0.3831	0.2241	0.5021

Table 2: The average p-values obtained using bottleneck, GH, KS, maximum gap, and ETL based statistics for various pairs of distributions considered for drawing edge weights of networks. There were 6 networks in each group for all the scenarios. The p-values smaller than 0.01 indicate that the corresponding method can identify network differences at a 99% confidence level.

Distribution	Bottleneck	GH	KS(β_0)	KS(β_1)	Maximum gap	**ETL**
$\text{Beta}(1,1)$ vs. $\text{Beta}(5,2)$	0.0029	0.0032	0.0879	0.2984	0.0020	0.0021
$\text{Beta}(1,1)$ vs. $\text{Beta}(1,5)$	0.0039	0.1391	0.3412	0.1333	0.0032	0.0028
$\text{Beta}(5,2)$ vs. $\text{Beta}(1,5)$	0.0001	0.8790	0.5854	0.4600	0.0015	0.0022
$\text{Beta}(1,1)$ vs. $\text{Beta}(1,1)$	0.8204	0.3911	0.9848	0.7357	0.0863	0.6430
$\text{Beta}(5,2)$ vs. $\text{Beta}(5,2)$	0.5272	0.0919	0.7677	0.6115	0.3785	0.7882
$\text{Beta}(1,5)$ vs. $\text{Beta}(1,5)$	0.4840	0.1640	0.4224	0.6654	0.1315	0.1526

analysis and not topological. Our topological methods are different. The use of the order statistic in quantifying topological difference between males and females is novel. This method identifies the impact of distribution differences in topological features. These specific results have not been observed before to best of our knowledge.

Table 3: The average p-values obtained using Wilcoxon rank sum test on the areas under β_0 curves for various pairs of distributions considered for drawing edge weights of networks. Here, the columns 6 networks, 8 networks, 10 networks, and 12 networks indicate the number of networks that we considered for both the groups. The p-values smaller than 0.01 indicate that our method can identify network differences at a 99% confidence level.

Distribution	6 networks	8 networks	10 networks	12 networks
Beta$(1,1)$ vs. Beta$(5,2)$	0.0022	1.5540×10^{-4}	1.8365×10^{-4}	3.6585×10^{-5}
Beta$(1,1)$ vs. Beta$(1,5)$	0.0022	1.5540×10^{-4}	1.8267×10^{-4}	3.6585×10^{-5}
Beta$(5,2)$ vs. Beta$(1,5)$	0.0022	1.5540×10^{-4}	1.8267×10^{-4}	3.6585×10^{-5}
Beta$(1,1)$ vs. Beta$(1,1)$	0.9372	0.2345	0.2413	0.4025
Beta$(5,2)$ vs. Beta$(5,2)$	0.8182	0.1605	0.6776	0.7075
Beta$(1,5)$ vs. Beta$(1,5)$	0.2403	0.6454	0.7913	0.1572

References

[Adler et al., 2010] Adler, R., Bobrowski, O., Borman, M., Subag, E., and Weinberger, S. (2010). Persistent homology for random fields and complexes. In *Borrowing strength: theory powering applications–a Festschrift for Lawrence D. Brown*, pages 124–143. Institute of Mathematical Statistics.

[Anand and Chung, 2021] Anand, D. V. and Chung, M. K. (2021). Hodge-laplacian of brain networks and its application to modeling cycles. *arXiv preprint arXiv:2110.14599*.

[Arslan et al., 2018] Arslan, S., Ktena, S., Makropoulos, A., Robinson, E., Rueckert, D., and Parisot, S. (2018). Human brain mapping: A systematic comparison of parcellation methods for the human cerebral cortex. *NeuroImage*, 170:5–30.

[Bauer, 2021] Bauer, U. (2021). Ripser: efficient computation of vietoris–rips persistence barcodes. *Journal of Applied and Computational Topology*, 5(3):391–423.

[Bishop, 2006] Bishop, C. (2006). *Pattern recognition and machine learning*. springer.

[Bobrowski and Kahle, 2018] Bobrowski, O. and Kahle, M. (2018). Topology of random geometric complexes: a survey. *Journal of applied and Computational Topology*, 1(3):331–364.

[Bollobás and Béla, 2001] Bollobás, B. and Béla, B. (2001). *Random graphs*. Number 73. Cambridge university press.

[Bubenik, 2015] Bubenik, P. (2015). Statistical topological data analysis using persistence landscapes. *Journal of Machine Learning Research*, 16:77–102.

[Chung et al., 2019a] Chung, M., Lee, H., DiChristofano, A., Ombao, H., and Solo, V. (2019a). Exact topological inference of the resting-state brain networks in twins. *Network Neuroscience*, 3:674–694.

[Chung et al., 2020] Chung, M., Smith, A., and Shiu, G. (2020). Reviews: Topological distances and losses for brain networks. *arXiv e-prints*, pages arXiv–2102.08623.

[Chung et al., 2019b] Chung, M., Xie, L., Huang, S.-G., Wang, Y., Yan, J., and Shen, L. (2019b). Rapid acceleration of the permutation test via transpositions. In *International Workshop on Connectomics in Neuroimaging*, volume 11848, pages 42–53. Springer.

[Conover, 1980] Conover, W. (1980). *Practical Nonparametric Statistics*. Wiley, New York.

[Desikan et al., 2006] Desikan, R., Ségonne, F., Fischl, B., Quinn, B., Dickerson, B., Blacker, D., Buckner, R., Dale, A., Maguire, R., Hyman, B., Marilyn, S., and Ronald, J. (2006). An automated labeling system for subdividing the human cerebral cortex on mri scans into gyral based regions of interest. *NeuroImage*, 31:968–980.

[Dirkx et al., 2016] Dirkx, M. F., den Ouden, H., Aarts, E., Timmer, M., Bloem, B. R., Toni, I., and Helmich, R. C. (2016). The cerebral network of parkinson's tremor: an effective connectivity fmri study. *Journal of Neuroscience*, 36(19):5362–5372.

[Edmonds and Karp, 1972] Edmonds, J. and Karp, R. (1972). Theoretical improvements in algorithmic efficiency for network flow problems. *Journal of the ACM (JACM)*, 19:248–264.

[Erdös and Rényi, 1961] Erdös, P. and Rényi, A. (1961). On the evolution of random graphs. *Bull. Inst. Internat. Statist*, 38:343–347.

[Fasy et al., 2014] Fasy, B. T., Kim, J., Lecci, F., and Maria, C. (2014). Introduction to the R package TDA. *arXiv preprint arXiv:1411.1830*.

[Fornito et al., 2016] Fornito, A., Zalesky, A., and Bullmore, E. (2016). *Fundamentals of Brain Network Analysis*. Academic Press, New York.

[Frieze and Karoński, 2016] Frieze, A. and Karoński, M. (2016). *Introduction to random graphs*. Cambridge University Press.

[Gayet and Welschinger, 2014] Gayet, D. and Welschinger, J.-Y. (2014). Lower estimates for the expected betti numbers of random real hypersurfaces. *Journal of the London Mathematical Society*, 90(1):105–120.

[Gibbons and Chakraborti, 2011] Gibbons, J. D. and Chakraborti, S. (2011). *Nonparametric Statistical Inference*. Chapman & Hall/CRC Press.

[Gilbert, 1959] Gilbert, E. N. (1959). Random graphs. *The Annals of Mathematical Statistics*, 30(4):1141–1144.

[Giunti et al., 2021] Giunti, B., Houry, G., and Kerber, M. (2021). Average complexity of matrix reduction for clique filtrations. *arXiv preprint arXiv:2111.02125*.

[Giusti et al., 2016] Giusti, C., Ghrist, R., and Bassett, D. S. (2016). Two's company, three (or more) is a simplex. *Journal of computational neuroscience*, 41(1):1–14.

[Hagmann et al., 2007] Hagmann, P., Kurant, M., Gigandet, X., Thiran, P., Wedeen, V., Meuli, R., and Thiran, J. (2007). Mapping human whole-brain structural networks with diffusion MRI. *PLoS One*, 2(7):e597.

[Horwitz et al., 2005] Horwitz, B., Warner, B., Fitzer, J., Tagamets, M.-A., Husain, F. T., and Long, T. W. (2005). Investigating the neural basis for functional and effective connectivity. application to fmri. *Philosophical Transactions of the Royal Society B: Biological Sciences*, 360(1457):1093–1108.

[Ignacio and Darcy, 2019] Ignacio, P. S. P. and Darcy, I. K. (2019). Tracing patterns and shapes in remittance and migration networks via persistent homology. *EPJ Data science*, 8(1):1.

[Janson et al., 2011] Janson, S., Rucinski, A., and Luczak, T. (2011). *Random graphs*. John Wiley & Sons.

[Jordan, 1999] Jordan, M. (1999). *Learning in graphical models*. MIT press.

[Kahle, 2007] Kahle, M. (2007). The neighborhood complex of a random graph. *Journal of Combinatorial Theory, Series A*, 114:380–387.

[Kahle, 2011] Kahle, M. (2011). Random geometric complexes. *Discrete & Computational Geometry*, 45:553–573.

[Kahle and Meckes, 2013] Kahle, M. and Meckes, E. (2013). Limit theorems for Betti numbers of random simplicial complexes. *Homology, Homotopy and Applications*, 15:343–374.

[Kannan et al., 2019] Kannan, H., Saucan, E., Roy, I., and Samal, A. (2019). Persistent homology of unweighted complex networks via discrete Morse theory. *Scientific reports*, 9:1–18.

[Kerber et al., 2017] Kerber, M., Morozov, D., and Nigmetov, A. (2017). Geometry helps to compare persistence diagrams. *Journal of Experimental Algorithmics*, 22.

[Koscik et al., 2009] Koscik, T., O'Leary, D., Moser, D., Andreasen, N., and Nopoulos, P. (2009). Sex differences in parietal lobe morphology: relationship to mental rotation performance. *Brain and cognition*, 69:451–459.

[Kwitt et al., 2015] Kwitt, R., Huber, S., Niethammer, M., Lin, W., and Bauer, U. (2015). Statistical topological data analysis-a kernel perspective. *Advances in neural information processing systems*, 28.

[Lee et al., 2011] Lee, H., Chung, M., Kang, H., Kim, B.-N., and Lee, D. (2011). Computing the shape of brain networks using graph filtration and Gromov-Hausdorff metric. *MICCAI, Lecture Notes in Computer Science*, 6892:302–309.

[Liu et al., 2016] Liu, T., Seyedhosseini, M., and Tasdizen, T. (2016). Image segmentation using hierarchical merge tree. *IEEE transactions on image processing*, 25(10):4596–4607.

[Morozov and Weber, 2013] Morozov, D. and Weber, G. (2013). Distributed merge trees. In *Proceedings of the 18th ACM SIGPLAN symposium on Principles and practice of parallel programming*, pages 93–102.

[Murai et al., 2001] Murai, T., Nakata, M., and Sato, Y. (2001). A note on filtration and granular reasoning. In *Annual Conference of the Japanese Society for Artificial Intelligence*, pages 385–389. Springer.

[Newman et al., 2001] Newman, M., Strogatz, S., and Watts, D. (2001). Random graphs with arbitrary degree distributions and their applications. *Physical Review E*, 64:26118.

[Nguyen et al., 2020] Nguyen, M., Aktas, M., and Akbas, E. (2020). Bot detection on social networks using persistent homology. *Mathematical and Computational Applications*, 25(3):58.

[Nigmetov and Morozov, 2019] Nigmetov, A. and Morozov, D. (2019). Local-global merge tree computation with local exchanges. In *Proceedings of the International Conference for High Performance Computing, Networking, Storage and Analysis*, pages 1–13.

[Obayashi, 2018] Obayashi, I. (2018). Volume-optimal cycle: Tightest representative cycle of a generator in persistent homology. *SIAM Journal on Applied Algebra and Geometry*, 2(4):508–534.

[O'Neil et al., 1996] O'Neil, P., Cheng, E., Gawlick, D., and O'Neil, E. (1996). The log-structured merge-tree (lsm-tree). *Acta Informatica*, 33(4):351–385.

[Park et al., 2018] Park, H.-J., Friston, K. J., Pae, C., Park, B., and Razi, A. (2018). Dynamic effective connectivity in resting state fmri. *NeuroImage*, 180:594–608.

[Petri et al., 2014] Petri, G., Expert, P., Turkheimer, F., Carhart-Harris, R., Nutt, D., Hellyer, P., and Vaccarino, F. (2014). Homological scaffolds of brain functional networks. *Journal of The Royal Society Interface*, 11:20140873.

[Petri et al., 2013] Petri, G., Scolamiero, M., Donato, I., and Vaccarino, F. (2013). Topological strata of weighted complex networks. *PloS one*, 8(6):e66506.

[Piangerelli et al., 2020] Piangerelli, M., Maestri, S., and Merelli, E. (2020). Visualising 2-simplex formation in metabolic reactions. *Journal of Molecular Graphics and Modelling*, 97:107576.

[Reimann et al., 2017] Reimann, M. W., Nolte, M., Scolamiero, M., Turner, K., Perin, R., Chindemi, G., Dłotko, P., Levi, R., Hess, K., and Markram, H. (2017). Cliques of neurons bound into cavities provide a missing link between structure and function. *Frontiers in computational neuroscience*, page 48.

[Rieck et al., 2021a] Rieck, J., Baracchini, G., Nichol, D., Abdi, H., and Grady, C. (2021a). Dataset of functional connectivity during cognitive control for an adult lifespan sample. *Data in Brief*, 39:107573.

[Rieck et al., 2021b] Rieck, J., Baracchini, G., Nichol, D., Abdi, H., and Grady, C. (2021b). Reconfiguration and dedifferentiation of functional networks during cognitive control across the adult lifespan. *Neurobiology of Aging*, 106:80–94.

[Rubin et al., 2017] Rubin, L., Yao, L., Keedy, S., Reilly, J., Bishop, J., Carter, C., Pournajafi-Nazarloo, H., Drogos, L., Tamminga, C., and Pearlson, G. (2017). Sex differences in associations of arginine vasopressin and oxytocin with resting-state functional brain connectivity. *Journal of neuroscience research*, 95:576–586.

[Salepci and Welschinger, 2018] Salepci, N. and Welschinger, J.-Y. (2018). Tilings, packings and expected betti numbers in simplicial complexes. *arXiv preprint arXiv:1806.05084*.

[Samardzic et al., 2020] Samardzic, N., Qiao, W., Aggarwal, V., Chang, M.-C. F., and Cong, J. (2020). Bonsai: High-performance adaptive merge tree sorting. In *2020 ACM/IEEE 47th Annual International Symposium on Computer Architecture (ISCA)*, pages 282–294. IEEE.

[Schlösser et al., 2003] Schlösser, R., Gesierich, T., Kaufmann, B., Vucurevic, G., Hunsche, S., Gawehn, J., and Stoeter, P. (2003). Altered effective connectivity during working memory performance in schizophrenia: a study with fmri and structural equation modeling. *Neuroimage*, 19(3):751–763.

[Sears and Ramakrishnan, 2012] Sears, R. and Ramakrishnan, R. (2012). blsm: a general purpose log structured merge tree. In *Proceedings of the 2012 ACM SIGMOD International Conference on Management of Data*, pages 217–228.

[Sharathkumar and Agarwal, 2012] Sharathkumar, R. and Agarwal, P. (2012). Algorithms for the transportation problem in geometric settings. In *Proceedings of the twenty-third annual ACM-SIAM symposium on Discrete Algorithms*, pages 306–317. SIAM.

[Solo et al., 2018] Solo, V., Poline, J., Lindquist, M., Simpson, S., Bowman, D., C. M., and Cassidy, B. (2018). Connectivity in fMRI: a review and preview. *IEEE Transactions on Medical Imaging*, page in press.

[Songdechakraiwut and Chung, 2020a] Songdechakraiwut, T. and Chung, M. (2020a). Dynamic topological data analysis for functional brain signals. *IEEE International Symposium on Biomedical Imaging Workshops*, pages 1–4.

[Songdechakraiwut and Chung, 2020b] Songdechakraiwut, T. and Chung, M. (2020b). Topological learning for brain networks. page arXiv:2012.00675.

[Songdechakraiwut and Chung, 2022] Songdechakraiwut, T. and Chung, M. (2022). Topological learning for brain networks. *Annals of Applied Statistics*, in press:arXiv:2012.00675.

[Stolz et al., 2016] Stolz, B. J., Harrington, H. A., and Porter, M. A. (2016). The topological" shape" of brexit. *arXiv preprint arXiv:1610.00752*.

[Stolz et al., 2017] Stolz, B. J., Harrington, H. A., and Porter, M. A. (2017). Persistent homology of time-dependent functional networks constructed from coupled time series. *Chaos: An Interdisciplinary Journal of Nonlinear Science*, 27(4):047410.

[Tadić et al., 2019] Tadić, B., Andjelković, M., and Melnik, R. (2019). Functional geometry of human connectomes. *Scientific reports*, 9(1):1–12.

[Topaz et al., 2015] Topaz, C., Ziegelmeier, L., and Halverson, T. (2015). Topological data analysis of biological aggregation models. *PLoS One*, page e0126383.

[Tzourio-Mazoyer et al., 2002] Tzourio-Mazoyer, N., Landeau, B., Papathanassiou, D., Crivello, F., Etard, O., Delcroix, N., Mazoyer, B., and Joliot, M. (2002). Automated

anatomical labeling of activations in spm using a macroscopic anatomical parcellation of the MNI MRI single-subject brain. *NeuroImage*, 15:273–289.

[Wigman, 2021] Wigman, I. (2021). On the expected betti numbers of the nodal set of random fields. *Analysis & PDE*, 14(6):1797–1816.

[Xu and Lindquist, 2015] Xu, Y. and Lindquist, M. (2015). Dynamic connectivity detection: an algorithm for determining functional connectivity change points in fMRI data. *Frontiers in Neuroscience*, 9:285.

[Zang et al., 2004] Zang, Y., Jiang, T., Lu, Y., He, Y., and Tian, L. (2004). Regional homogeneity approach to fmri data analysis. *Neuroimage*, 22:394–400.
